# Using time series vector features for annual cultivated land mapping: A trial in northern Henan, China

**DOI:** 10.1371/journal.pone.0272300

**Published:** 2022-08-09

**Authors:** Xiaoping Lu, Yushi Zhou, Xiangjun Zhang, Haikun Yu, Guosheng Cai

**Affiliations:** 1 Key Laboratory of Spatio-temporal Information and Ecological Restoration of Mines of Natural Resources of the People’s Republic of China, Henan Polytechnic University, Jiaozuo, China; 2 Henan Institute of Remote Sensing and Surveying and Mapping, Zhengzhou, China; University of Kurdistan Hewlêr, Kurdistan Region, IRAQ

## Abstract

Annual monitoring of the spatial distribution of cultivated land is important for maintaining the ecological environment, achieving a status quo of land resource management, and guaranteeing agricultural production. With the gradual development of remote sensing technology, it has become a common practice to obtain cultivated land boundary information on a large scale with the help of satellite Earth observation images. Traditional land use classification methods are affected by multiple types of land cover, which leads to a decrease in the accuracy of cultivated land mapping. In contrast, although the current advanced methods (such as deep learning) can obtain more accurate cultivated land mapping results than traditional methods, such methods often require the use of a massive amount of training samples, large computing power, and highly complex model tuning processes, increasing the cost of mapping and requiring the involvement of more professionals. This has hindered the promotion of related methods in mapping institutions. This paper proposes a method based on time series vector features (MTVF), which uses vector thinking to establish the features. The advantage of this method is that the introduction of vector features enlarges the differences between the different land cover types, which overcomes the loss of mapping accuracy caused by the influences of the spectra of different ground objects and ensures the calculation efficiency. Moreover, the MTVF uses a traditional method (random forest) as the classification core, which makes the MTVF less demanding than advanced methods in terms of the number of training samples. Sentinel-2 satellite images were used to carry out cultivated land mapping for 2020 in northern Henan Province, China. The results show that the MTVF has the potential to accurately identify cultivated land. Furthermore, the overall accuracy, producer accuracy, and user accuracy of the overall study area and four sub-study areas were all greater than 90%. In addition, the cultivated land mapping accuracy of the MTVF is significantly better than that of the maximum likelihood, support vector machine, and artificial neural network methods.

## Introduction

Cultivated land is one of the main types of land cover, and it is a key component of human food production. At present, the world’s cultivated land feeds more than seven billion people. Due to rapid population growth, the development of cultivated land far exceeds its carrying capacity. Therefore, monitoring the annual spatial distribution of cultivated land is an important prerequisite for protecting the ecological environment [1], establishing a new status quo in land resource management [2], and guaranteeing agricultural production [3–8]. The mastery of annual cultivated land mapping data has become an important research topic.

Satellite remote sensing technology has gradually become one of the main methods for cultivated land mapping due to its timeliness, low cost, and large-scale observation capabilities. At present, with the help of remote sensing observation archives, many global cultivated land mapping products and global land cover mapping products containing cultivated land map layers have been released, including the finer resolution observation and monitoring of global land cover (FROM–GLC) [9, 10], GlobeLand 30 [11], data and information system global land cover (DISCover) [12], and moderate resolution imaging spectroradiometer (MODIS) [13, 14] land cover products. However, the above products are only available for certain years (such as 2015 and 2020), and thus, they do not meet the requirements for annual cultivated land mapping. Furthermore, due to the limitations of the spatial scales of the existing global cultivated land mapping products, the definition of cultivated land often fails to take into account the local scale. Sustainable development research, food security, and other fields have created new requirements for higher resolution, high precision, and local cultivated land monitoring data.

There are various methods for cultivated land mapping based on remote sensing observation data, including traditional supervised classification models (e.g., the maximum likelihood method [15], support vector machines [16], and artificial neural networks [17]) and advanced methods (e.g., deep learning [18] and artificial immune networks [19]). However, regarding the cultivated land mapping process, the existing methods still encounter many limitations. For example, traditional methods are susceptible to the effects of multiple land cover types, resulting in a decrease in the accuracy of cultivated land surveying and mapping [20]; whereas advanced methods (e.g., deep learning) need to rely more on massive training samples, super-computing power, and complex model tuning [21, 22]. Establishing a simple but reliable method is the key to cultivated land mapping research.

The selection of data for cultivated land mapping is also important. One feasible idea is to use time series data. Many studies have shown that multi-spectral remote sensing images based on time series sequences are an effective means of large-scale, long-term, continuous agricultural remote sensing mapping [23, 24]. Multispectral remote sensing image data based on time series can overcome the influences of various factors, such as the weather, and can provide a data basis for continuous crop growth curve extraction [25, 26]. Time series analysis combined with the vegetation index is also an effective idea for cultivated land mapping. As the most widely used characteristic parameter to describe vegetation phenological changes [27], the vegetation index time series can reflect the dynamic changes in different crop types over time. The vegetation index based on time series multi-spectral remote sensing image data reflects the dynamic changes in different crop types over time.

Based on the above discussion, a method based on time series vector features (MTVF) is proposed in this article. The core of this method is to use a time series based on spectral and vegetation indices as a vector and to extract the vector features to distinguish the differences between cultivated land and other land cover types. The purposes of this approach are as follows. 1) This approach can widen the difference between other land cover types and cultivated land and reduce the impact of spectral overlap. 2) Compared with deep learning methods, the proposed method requires fewer training samples and less computing power, and thus, it can significantly improve the efficiency of cultivated land mapping. In this study, Sentinel-2 satellite data were used to generate a 10-m spatial resolution cultivated land map because these data have a short revisit period and contain rich red-edge band information. This method was applied to the study area in the northern part of Henan Province, China, to verify its applicability to cultivated land mapping. In addition, four sub-study areas located in different landscapes within the study area were established to evaluate the cultivated land maps. Finally, the MTVF was compared with three traditional supervised classification models (the maximum likelihood, support vector machine, and artificial neural network models) to evaluate the advantages of this method in cultivated land mapping.

## Study area

**Fig 1:**
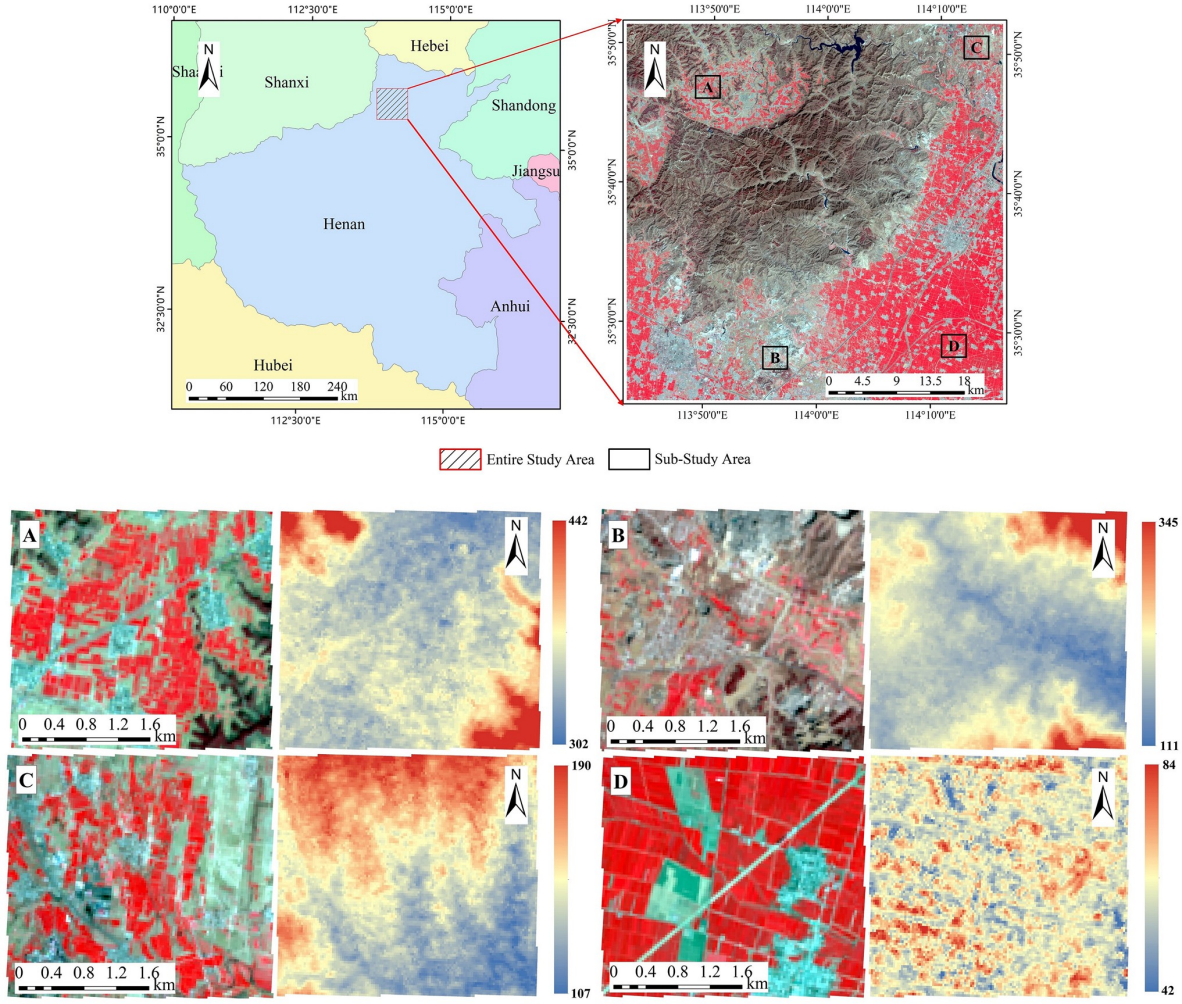
Study area. Note: The pseudo-color image (R: NIR, G: Red, B: Green) presented in the figure is a Landsat-8 image acquired on March 19, 2020 (https://landsat.visibleearth.nasa.gov/), this Landsat image is similar but not identical to the original image used in the study. The digital elevation image is an advanced spaceborne thermal emission and reflection radiometer (ASTER) global digital elevation model (GDEM) Version 3 image (https://earthdata.nasa.gov/learn/articles/new-aster-gdem), and the digital elevation imagery legend is in meters.

In this study, a 50 km × 50 km area in the northern part of Henan Province was selected as the study area (Fig 1). The selection of this location was mainly based on the following factors: (i) the diversity of the plant types, (ii) the complexity of the land cover, (iii) the presence of multiple types of topography, and (iv) the heterogeneous spatial distribution of the grain yield.

Henan Province, which is located in the central part of China, is one of the country’s major grain cultivation areas. According to official statistics [28], the total cultivated land area in Henan Province was about 14.68×10^6^ hectares in 2019, an increase of about 2.01% compared to that in 2012. The northern part of Henan Province has a warm, temperate, monsoon climate, with an average annual rainfall of 500–900 mm and an average annual sunshine duration of 1285.7–2292.9 h, which makes it suitable for the growth of a variety of crops. Agricultural production activities in this area are affected by many factors, such as the water resources [29], labor per unit area, and urbanization rate [30].

To evaluate the effectiveness of the cultivated land mapping, four sub-areas within the study area were selected. Sub-study area A (LZ) was located in Linzhou County. LZ was located in the Taihang Mountains, and it contained a large amount of abandoned land, as well as abundant grass, woodland, and other natural vegetation. This was the main reason that LZ was selected as a sub-study area. Sub-study area B (XX) was located in the city of Xinxiang. The reason for choosing XX was mainly to consider the impact of open-pit mines on cultivated land. Sub-study area C (HB) was located in the city of Hebi. The natural grassland in HB was very lush, and the distribution of the cultivated land landscape was relatively fragmented. Experimentation in this area helped us to analyze the ability of the algorithm to separate grassland and cultivated land. Sub-study area D (WH) was located in Weihui County and contained large grape plantations. WH was selected to analyze the ability of the algorithm to strip orchards (grapes) in the process of cultivated land mapping. All of the sub-study areas were delimited by a 3 km × 3 km rectangle, and the winter wheat-summer corn rotation pattern was dominant in the four sub-study areas.

## Data preparation

### Satellite data

Sentinel-2 is a polar-orbit, high-resolution, multi-spectral imaging mission for terrestrial monitoring. It consists of two satellites, Sentinel-2A and Sentinel-2B, which are each equipped with a multi-spectral imager (MSI). Sentinel-2 satellite data includes data for 13 spectral bands, ranging from visible and near-infrared light to short-wave infrared light, with a ground resolution of up to 10 m (Table 1). Since the Sentinel-2 satellite can provide data in three spectral bands within the red-edge range and the data update cycle is 5 days (two satellites for monitoring), the Sentinel-2 satellite data have advantages relevant to vegetation time series monitoring. All of the data collected by the Sentinel-2 satellite can be downloaded from the European Space Agency (ESA) Copernicus Open Access Hub (https://scihub.copernicus.eu/dhus/#/home).

**Table 1:** Spectral bands of the Sentinel-2 sensors (Sentinel-2A and Sentinel-2B).

Sentinel-2 Bands	S2A		S2B		Spatial resolution (m)
	Central wavelength (nm)	Bandwidth (nm)	Central wavelength (nm)	Bandwidth (nm)	
Band 1—Coastal aerosols	442.7	21	442.2	21	60
Band 2—Blue	492.4	66	492.1	66	10
Band 3—Green	559.8	36	559.0	36	10
Band 4—Red	664.6	31	664.9	31	10
Band 5—Vegetation red edge	704.1	15	703.8	16	20
Band 6—Vegetation red edge	740.5	15	739.1	15	20
Band 7—Vegetation red edge	782.8	20	779.7	20	20
Band 8—NIR^a^	832.8	106	832.9	106	10
Band 8A—Vegetation red edge	864.7	21	864.0	22	20
Band 9—Water vapor	945.1	20	943.2	21	60
Band 11—SWIR^b^	1373.5	31	1376.9	30	60
Band 12—SWIR	1613.7	91	1610.4	94	20

^a^ NIR refers to the near infrared band.

^b^ SWIR refers to the shortwave infrared band.

Since the main planting pattern in the study area was the rotation of winter wheat and summer corn, October (the winter wheat was sown in the study area during this month) was selected as the annual time node. Based on this idea, we selected 12 scenes of Sentinel-2 images from October 2019 to September 2020 (Table 2). The scenes were selected to be as cloudless, fogless, and evenly distributed in each month as possible. These image data served as the basis for constructing the vegetation index time series.

**Table 2:** Sentinel-2 image acquisition dates.

Image acquisition date	Satellite	Image acquisition date	Satellite
10/30/2019	S2B	5/17/2020	S2B
11/14/2019	S2A	6/6/2020	S2B
12/29/2019	S2B	7/6/2020	S2B
1/28/2020	S2B	8/30/2020	S2A
2/17/2020	S2B	9/4/2020	S2B
3/18/2020	S2B	9/19/2020	S2A
4/17/2020	S2B		

It should be noted that Sentinel-2 images acquired in the middle of each month were selected to ensure the uniform distribution of the time series. Still, the acquisition times of some of the images changed to avoid excessive cloud cover. For example, the acquisition times of the October and December 2019 images were later in the month, and the acquisition times of the June and July 2020 images were in the early part of the month. The L2A level Sentinel-2 images were obtained from the ESA Copernicus Open Access Hub and had already been subjected to official ESA geometric and atmospheric corrections (see https://sentinel.esa.int/web/sentinel/user-guides/sentinel-2-msi/processing-levels/level -2), so they could be directly used to calculate the vegetation index.

### Collection of reference data

#### Reference land cover classes in study area

To investigate the algorithm’s ability to separate cultivated land from other land cover types, based on the results of field investigations and the land cover classification system developed by the Chinese Academy of Sciences, the main land cover types in the study area were determined.

- Cultivated land: This refers to the land on which crops are grown, including food crops and some vegetables. It should be noted that fruit plantations were not included in this category, because in the study area, orchards were not the main form of agriculture and were also under the jurisdiction of the local forestry management department.
- Woodland: This refers to forestry land where trees, shrubs, bamboo, and orchards grow.
- Grassland: This refers to land dominated by natural herbaceous plants. The study area did not contain artificial grassland, so it was not considered.
- Water: This refers to natural terrestrial water area and land used for water conservancy facilities, such as rivers, artificial canals, lakes, ponds, and pools.
- Artificial construction land: This refers to urban, rural, industrial, mining, and residential land.
- Bare land: This refers to land and rocks with vegetation coverage of less than 5%. In the study area, rock exposed by mining was also included in this type.

#### Collection of training and validation samples

**Fig 2:**
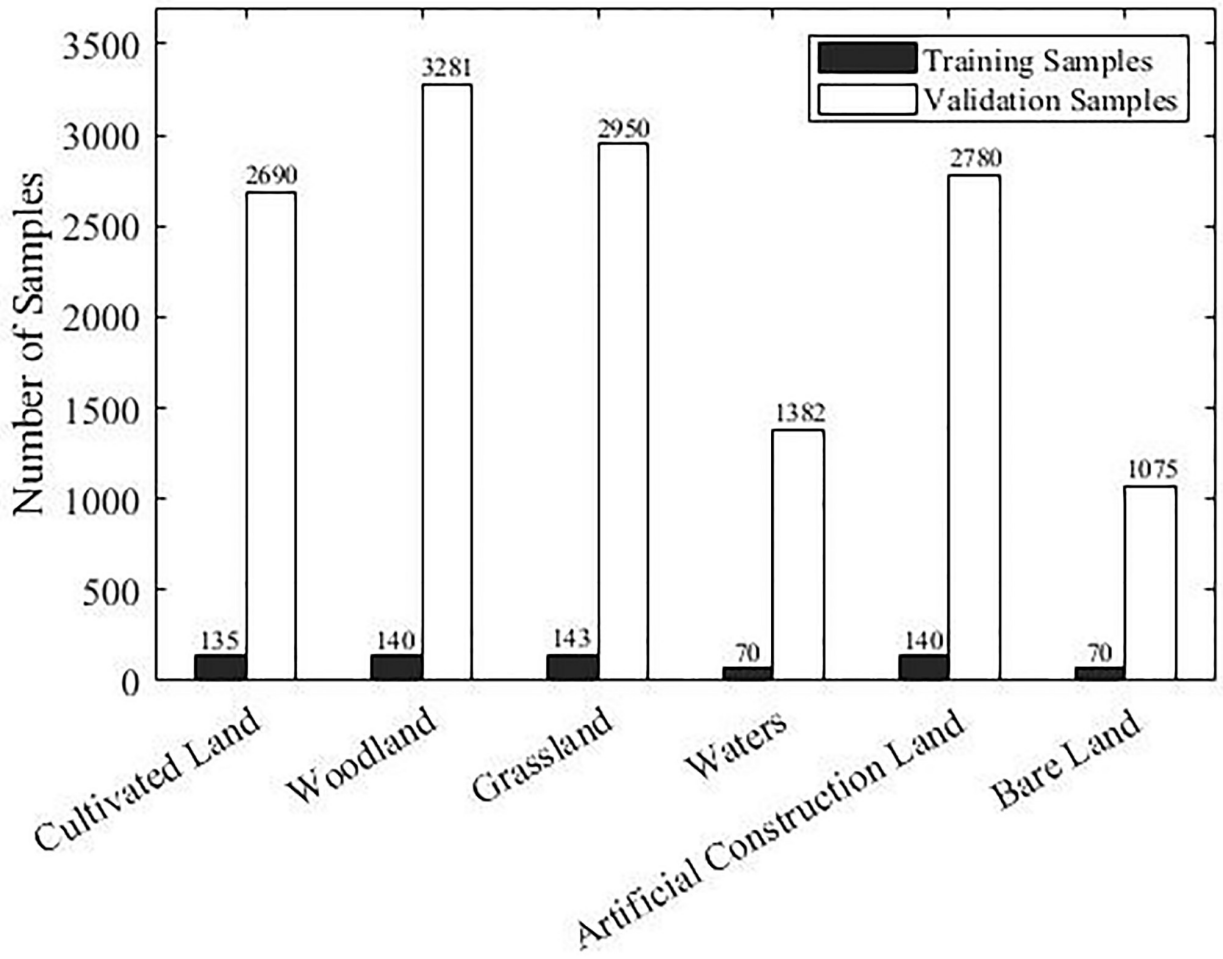
Numbers of training samples and validation samples for each type of land cover.

We collected training and validation samples in the study area in the form of pixels. In order to obtain the above information, we combined a field survey (May 2021) and a visual inspection of very-high-resolution (VHR) images from Google Earth and unmanned aerial vehicle (UAV) field measurements, the VHR image from Google Earth was acquired on December 31, 2019, and the VHR images from the UAV were acquired on May 11–14, 2021. December and May were chosen because of the agricultural characteristics of the study area. December is the emergence period of winter wheat, so it is possible to effectively distinguish cultivated land from other vegetation in this month. May is the maturity period of wheat, so it is convenient to screen non-vegetation covered land types in this month. During the field investigations, the coordinates and attribute information about the ground objects were mainly recorded using a handheld global positioning system (GPS). To overcome the inaccuracy caused by the different years, the samples were screened by manually comparing the 2021 VHR images acquired using the UAV and the Sentinel-2 images to ensure that the attributes of the sample points were consistent with the actual land cover types in 2020. In the study area, 698 and 14,158 well disturbed pixels were used as training samples and verification samples, respectively (Fig 2). Although including more training samples would have had a positive effect on the remote sensing supervised classification calculations, we used fewer training samples in order to verify the advantages of our algorithm. For supervised classification, the general rule is that the number of training samples per class should be 10–30 times the input image band [31–33]. Therefore, in this paper, ten times the number of bands of the Sentinel-2 satellite imagery was used as the reference value. Training samples for each surface coverage category were selected from the collected samples, and the remaining samples were used as the verification samples. However, the distributions of the water and bare land in the study area were limited, so only 70 pixels were selected as training samples for each of these two land cover types.

In order to verify the accuracy of the results for the four sub-study areas, several pixels were collected from each sub-study area as verification samples. According to the ground truth, these pixels were labeled as 1 (cultivated land) or 0 (non-cultivated land). The distribution of the samples is shown in Table 3.

**Table 3:** Numbers of verification samples in the four sub-study areas (pixels).

Land cover classification	Cultivated land	Non-cultivated land	Total
LZ	837	1044	1881
XX	153	1213	1366
HB	952	1969	2921
WH	2381	1552	3933

## Methods

### Methodological overview

**Fig 3:**
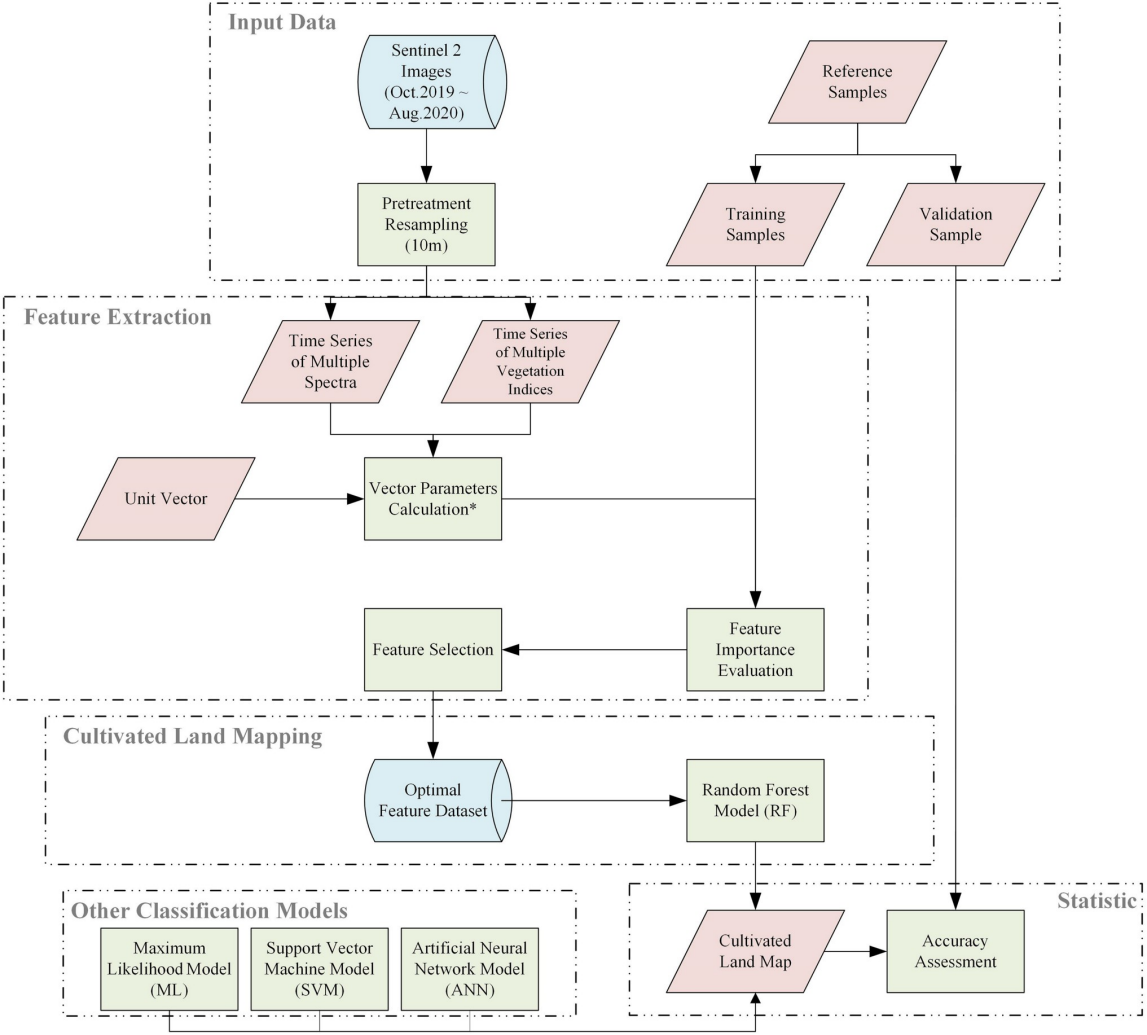
Work flow of the proposed method (the vector parameters included Cos, Dis, Max, Min, and Ran).

Fig 3 summarizes the method of annual cultivated land mapping using time series vector features. First, we extracted each band value from the Sentinel-2 satellite images with different acquisition times and calculated the 23 vegetation indices for the corresponding times. The selection of the vegetation indices mainly followed the principle that the Sentinel-2 image bands could be generated and verified in practice. The time series of multiple spectra and the time series of multiple vegetation indices (Table 4) were used as vectors for the subsequent calculations (see Vector Construction). Five parameters (i.e., the cosine, distance, maximum, minimum, and range of each vector) were calculated (see Feature Extraction). Then, we calculated the importance score of each parameter (based on the random forest model) to evaluate the contribution of each parameter to the cultivated land mapping and to provide a thorough analysis and discussion. By sorting and grouping the importance scores of the parameters and calculating the performances of the different groupings in terms of the random forest prediction accuracy and the out-of-bag error, several optimal parameters that could be used in the calculations for the cultivated land mapping in the study area were determined. The number of trees was also determined in this manner (see Random Forest Model). The training samples and optimal parameters were introduced into the random forest classification algorithm to generate the cultivated land map layer in the next step. Finally, the classification accuracy of the cultivated land map was evaluated through the verification sample and compared with the results obtained using other algorithms (i.e., the maximum likelihood, support vector machine, and artificial neural network methods; see Other Classification Models and Accuracy Assessment).

**Table 4:** Selected vegetation indices.

Vegetation index	Reference	Vegetation index	Reference
Soil Adjusted Vegetation Index (SAVI)	[34]	Atmospherically Resistant Vegetation Index (ARVI)	[45]
Transformed Soil Adjusted Vegetation Index (TSAVI)	[35, 36]	Normalized Difference Index (NDI 45)	[46]
Modified Soil Adjusted Vegetation Index (MSAVI)	[37]	Meris Terrestrial Chlorophyll Index (MTCI)	[47]
Second Modified Soil Adjusted Vegetation Index (MSAVI 2)	[37]	Modified Chlorophyll Absorption Ratio Index (MCARI)	[48]
Difference Vegetation Index (DVI)	[38]	Sentinel-2 Red-Edge Position Index (S2REP)	[49]
Ratio Vegetation Index (RVI)	[39]	Inverted Red-Edge Chlorophyll Index (IECI)	[50]
Perpendicular Vegetation Index (PVI)	[40]	Pigment Specific Simple Ratio (PSSR)	[51]
Infrared Percentage Vegetation Index (IPVI)	[41]	Normalized Difference Vegetation Index (NDVI)	[52]
Weighted Difference Vegetation Index (WDVI)	[42]	Modified Red Edge Normalized Difference Vegetation Index (NDVI_705_)	[53]
Transformed Normalized Difference Vegetation Index (TNDVI)	[38]	Enhanced Vegetation Index (EVI)	[54]
Green Normalized Difference Vegetation Index (GNDVI)	[43]	2-Band Enhanced Vegetation Index (EVI2)	[55]
Global Environmental Monitoring Index (GEMI)	[44]		

### Vector construction

The parameters used to build the vector included the Sentinel-2 satellite band value and vegetation indices. The reflectivity characteristics of the different features in each wavelength range were dissimilar. Thus, the 13 bands of the Sentinel-2 satellite were used as the radiation parameters to establish the vector, and these data were normalized. Furthermore, a total of 23 vegetation indices (Table 4) were selected as the vegetation index parameters for the cultivated land mapping. The selection of the vegetation indices mainly followed two rules: 1.) they can be produced from the bands of the Sentinel-2 satellite images; and 2.) they have been verified in practice. These vegetation indices were calculated using the ESA Sentinel Application Platform (SNAP) software (http://step.esa.int/main/toolboxes/snap/). All of the radiation parameters and vegetation index parameters were constructed as vectors in the form of time series and can be expressed as follows:

$$\vec{V_{p}}=\left[\begin{array}{c} p_{Oct}\\ \begin{array}{c} p_{Nov}\\ p_{Dec}\\ \vdots \end{array}\\ p_{Sept} \end{array}\right],$$ (1)

where $\vec{V_{p}}$ is the time series vector of parameter p from October 2019 to September 2020.

### Feature extraction

Vector features can be used as significant parameters for remote sensing image classification, including the vector angle [56, 57], vector distance [58], and extreme value [59–61]. Therefore, we established five parameters: the cosine, distance, maximum, minimum, and range based on the vector $\vec{V_{p}}$.

By analyzing each time series vector, three features were established:

$${Max}_{p}=\text{max}\;\left(p_{Oct},p_{Nov},\ldots ,p_{Spet}\right),$$ (2)

$${Min}_{p}=\text{min}\;\left(p_{Oct},p_{Nov},\ldots ,p_{Spet}\right),$$ (3)

$${Ran}_{p}={Max}_{p}-{Min}_{p},$$ (4)

where *Max*_*p*_, *Min*_*p*_, and *Ran*_*p*_ are the maximum, minimum, and range of time series vector $\vec{V_{p}}$, respectively. To extract the characteristics of each time series vector, a unit vector was established as a reference:

$$\vec{V_{0}}=\left[\begin{array}{c} 1\\ \begin{array}{c} 1\\ 1\\ \vdots \end{array}\\ 1 \end{array}\right].$$ (5)

Then, two features were established:

$${Cos}_{p}=\frac{\sum \vec{V_{0}}\vec{V_{p}}}{\sqrt{\sum {\left(\vec{V_{0}}\right)}^{2}\sum {\left(\vec{V_{p}}\right)}^{2}}},$$ (6)

$${Dis}_{p}=\sqrt{\sum {\left(\vec{V_{0}}-\vec{V_{p}}\right)}^{2}},$$ (7)

where *Cos*_*p*_ and *Dis*_*p*_ are the cosine and distance of the angle between the timing vector $\vec{V_{p}}$ and the reference vector $\vec{V_{0}}$, respectively.

### Random forest model

The random forest (RF) model is a machine learning classifier that combines multiple decision trees [62]. The random forest classification process can be described as follows:

**Step 1**. The bootstrap sampling method is used to randomly select training samples with replacement.
**Step 2**. Step 2. Set the corresponding decision tree model for each training sample and continue to split until all the training samples of the node are of the same type.
**Step 3**. The generated multiple decision trees are formed into a random forest, and the optimal classification is determined according to the voting probability.

In this study, the importance score, out-of-bag error, prediction accuracy, and classification calculations were performed using the random forest model. The importance score was used to determine the contribution of each feature to the cultivated land mapping. The out-of-bag error and prediction accuracy were used to select the feature set and to determine the number of trees in the random forest. All of the calculations were based on the Scikit-learn [63] machine learning algorithm library built in the Python language.

#### Importance score

The Gini coefficient generated by the random forest algorithm was used to compare the contributions of the individual features to the cultivated land mapping. The classification and regression tree method was integrated with the RF to provide the Gini coefficient for the next split. Thus, the importance of each feature was expressed as follows:

$$I_{f}=\sum_{i=1}^{n}\sum_{j=1}^{k}\Delta G,$$ (8)

$$\Delta G=G-\left(G_{L}+G_{R}\right),$$ (9)

$$S_{f}=\frac{I_{f}}{I},$$ (10)

where *G* is the Gini coefficient before the split, and *G*_*L*_ and *G*_*R*_ are the Gini coefficients of the left and right branches after the split. It is assumed that there are a total of *n* trees in the forest that use feature *f*, and the number of splits of each tree is *k*. *I*_*f*_ is the importance, and *I* is the sum of the importance of all of the features. *S*_*f*_ is the importance score of feature *f*, which is the normalized value of *I*_*f*_.

#### Out-of-bag error

A significant advantage of the random forest model is that it can build an unbiased estimate of the error internally, which is called the out-of-bag error (oob). For each tree, the randomly selected samples were approximately 63.2% of the total number of samples, and the remaining approximately 36.8% of the samples were designated as the oob samples of this tree. The oob of a random forest is the mean value of the oob of all of the trees in the model.

### Feature selection

An excessive number of features leads to higher computational costs and redundancy, so it is necessary to select appropriate features to participate in the next step of the calculation. In this study, the feature selection mainly included three steps. First, all of the parameters were sorted according to their importance scores to obtain their distribution characteristics. Then, all of the parameters were grouped according to their distribution characteristics to obtain several feature groups. Finally, all of the feature groups were input into the random forest model, the performances of the prediction accuracy and oob were calculated for different numbers of trees, and the group with the highest prediction accuracy and the smallest out-of-band error was selected as the feature group.

### Other classification models

To evaluate the cultivated land mapping model developed in this study, the maximum likelihood model, support vector machine model, and artificial neural network model were introduced for comparison. The same training and validation samples used in the MTVF were used in all of the traditional models to ensure the objectivity of the cultivated land mapping results of the comparison of the different models. The values of the parameters of each model were set within the range recommended by the developer because this is generally considered to be a safe practice [64].

#### Maximum likelihood model

The maximum likelihood model is one of the most widely used supervised classification models. It is a theoretical point estimation algorithm. The maximum likelihood model assumes that the distributions of the various types of data in each band are Gaussian, and each peak represents a unique feature category. Based on the training samples, the statistics of the normal distribution are obtained, and then, the probability of each pixel belonging to a different normal distribution is calculated. Finally, the pixel is classified into the category with the largest probability. The classification results of the maximum likelihood model have the advantages of stability, reliability, algorithm simplicity, high accuracy, and fast calculation times [15].

#### Support vector machine model

The concept of the support vector machine (SVM) was first proposed by Cortes and Vapnik [16]. It maps the vector to a higher dimension and implements classification by introducing a kernel function to map the sample data in the low-dimensional feature space to the high-dimensional feature space. In recent years, support vector machine models have been widely used for the segmentation, fusion, and classification of high-spatial-resolution images [65].

#### Artificial neural network model

The artificial neural network model is a non-parametric classification method with a good adaptability and a complex mapping capability. By mimicking the brain’s structure and functions, it realizes non-linear data pattern recognition and can effectively combine the spectral and textural features of images to improve the classification accuracy [17, 66, 67].

### Accuracy assessment

A confusion matrix was used to assess the accuracy of the cultivated land mapping. In this study, the accuracy of the producer (PA), the accuracy of the user (UA), the overall accuracy (OA), the kappa coefficient of variation (kappa), and the ground truth (by pixel) were chosen as the indices to measure the cultivated land mapping ability of each algorithm.

## Results

### Importance score of features

By calculating a total of 180 features, the 50 most important features are listed in Table 5. Among all of the features, Band 12 and Band 8A exhibited advantages in cultivated land mapping, with importance scores of 0.1723 and 0.1368, respectively. Among the top 10 parameters (importance scores of > 0.02), five of the parameters were based on the band value of the image, and the other five parameters were based on the vegetation indexes. The parameters characterized by the vector angle and the vector distance occupied dominant positions in the top 10 ranking of the importance. For the vegetation indexes, the normalized difference vegetation index (NDVI_705_, importance score of 0.0526) made the largest contribution to the cultivated land mapping, followed by the atmospherically resistant vegetation index (ARVI, importance score of 0.0516) and green normalized difference vegetation index (GNDVI, importance score of 0.0440). There were 26 features with importance scores greater than the average score (1/180 ≈ 0.0056).

**Table 5:** Top 50 features (from high to low) in terms of the importance score.

Rank	Feature	Importance score	Rank	Feature	Importance score
1	B12_Cos	0.1723	26	NDI 45_Dis	0.0058
2	B8A_Dis	0.1368	27	ARVI_Dis	0.0053
3	NDVI_705__Min	0.0526	28	IRECI_Cos	0.0052
4	ARVI_Cos	0.0516	29	REIP_Cos	0.0051
5	B7_Dis	0.0462	30	TNDVI_Ran	0.0051
6	GNDVI_Cos	0.0440	31	NDI 45_Ran	0.0050
7	PVI_Dis	0.0440	32	MCARI_Dis	0.0048
8	GEMI_Dis	0.0399	33	RVI_Cos	0.0048
9	B6_Ran	0.0347	34	B8_Max	0.0045
10	B11_Dis	0.0298	35	B11_Cos	0.0045
11	B9_Dis	0.0146	36	TSAVI_Ran	0.0042
12	B1_Min	0.0138	37	NDVI_705__Ran	0.0040
13	NDI 45_Cos	0.0135	38	MTCI_Max	0.0039
14	B1_Ran	0.0128	39	B8A_Max	0.0039
15	TSAVI_Dis	0.0126	40	B7_Cos	0.0038
16	B1_Dis	0.0124	41	B2_Min	0.0038
17	B4_Min	0.0116	42	MSAVI_Max	0.0038
18	B8_Dis	0.0114	43	B12_Ran	0.0036
19	TSAVI_Min	0.0111	44	MCARI_Min	0.0034
20	WDVI_Ran	0.0100	45	PVI_Ran	0.0032
21	B11_Min	0.0092	46	GNDVI_Dis	0.0030
22	B5_Dis	0.0070	47	B3_Max	0.0029
23	B1_Cos	0.0069	48	B1_Max	0.0029
24	MCARI_Ran	0.0067	49	PSSRA_Ran	0.0029
25	B12_Max	0.0064	50	B6_Cos	0.0028

### Feature selection results

**Fig 4:**
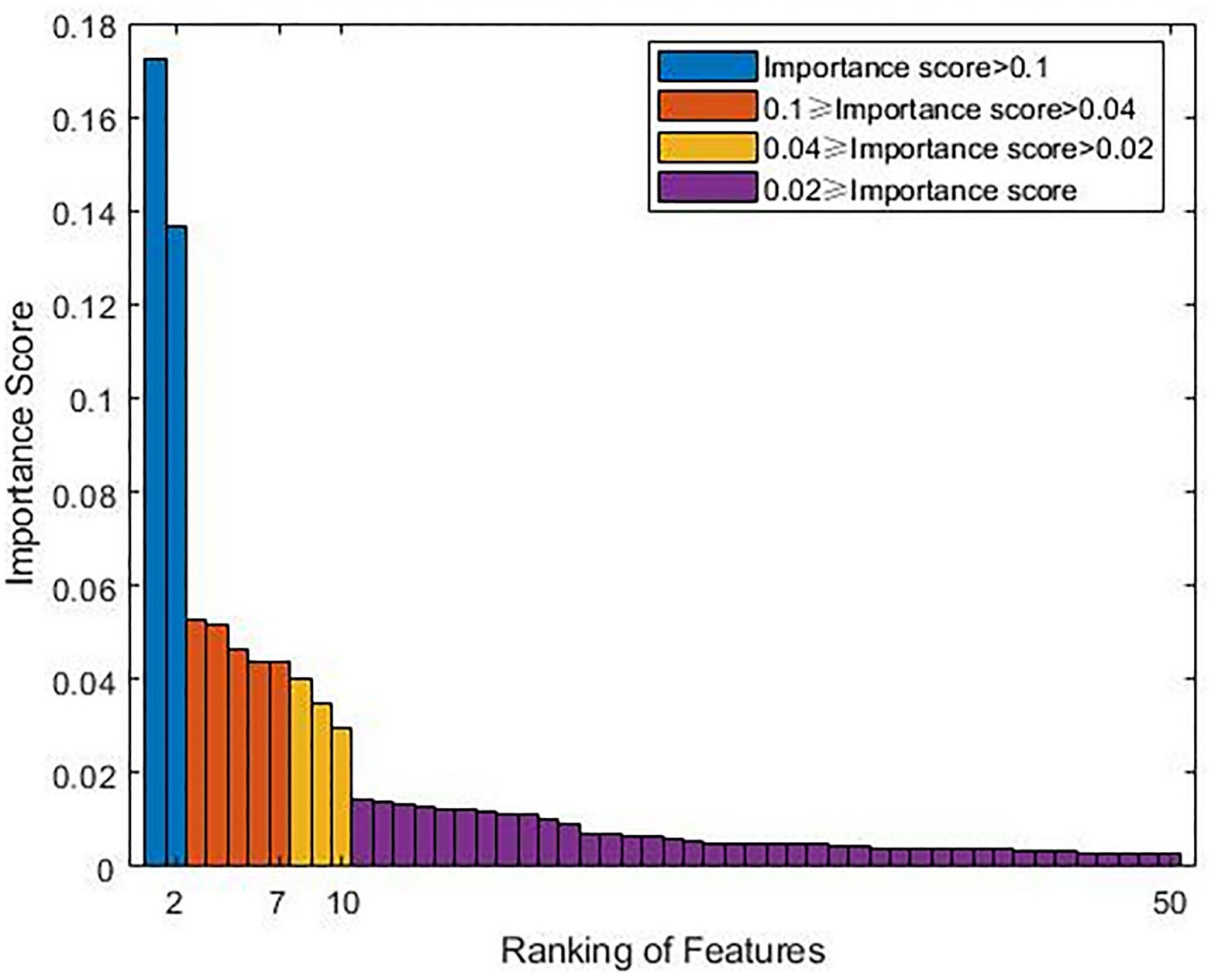
Bar graph of the top 50 features in terms of the importance score.

**Fig 5:**
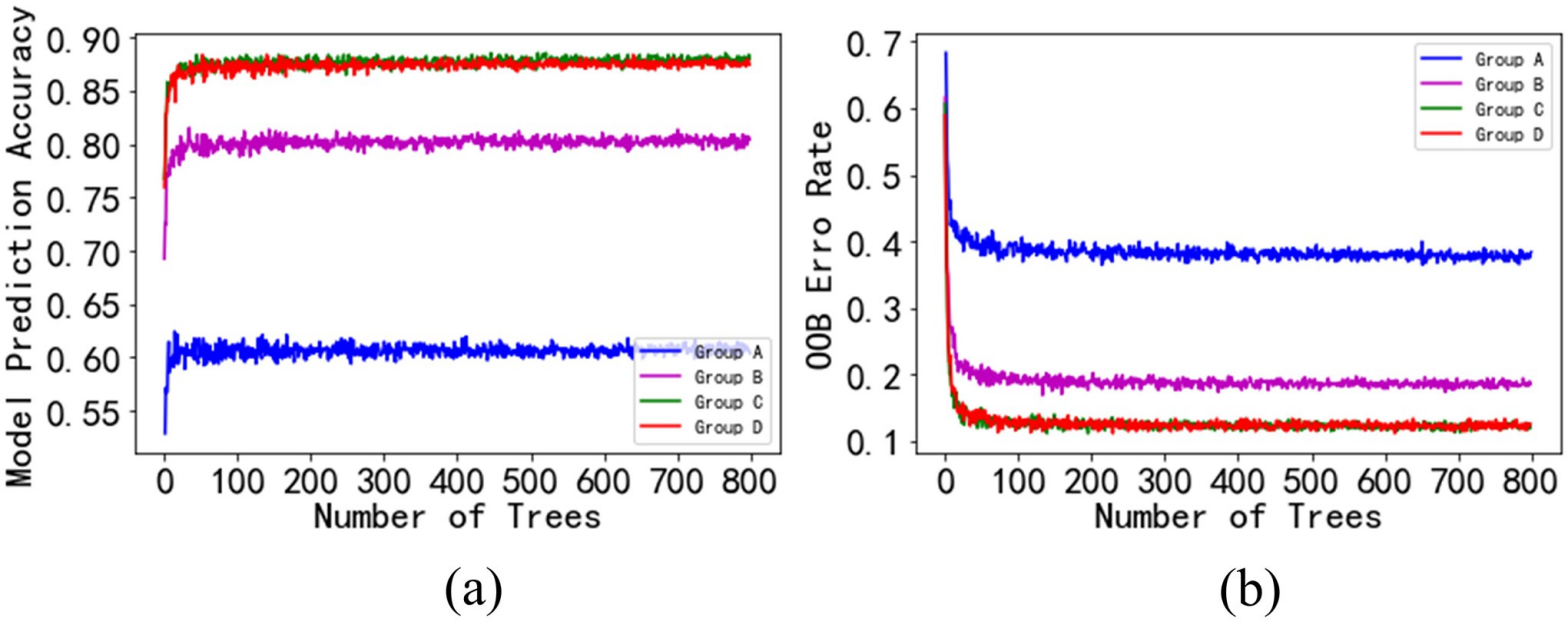
Influences of the different feature group inputs on the classification results of the random forest model: a) relationship between the model prediction accuracy and the number of trees; and b) relationship between the out-of-bag error and the number of trees.

The visualization of the importance score from high to low (Fig 4) shows that the importance scores exhibited three nodes: 0.1, 0.04, and 0.02. Based on this, four feature groups were established in this study: Group A (importance scores of > 0.1), Group B (importance scores of > 0.04), Group C (importance scores of > 0.02), and Group D (importance scores of > 0). The four feature groups were input into the random forest model to obtain the prediction accuracy and to determine the changes in the oob (Fig 5). The results revealed that Group C had the highest prediction accuracy and the lowest oob. Therefore, the features with importance scores of > 0.02 were selected, and the number of trees was set to 400.

### Accuracy assessment of cultivated land mapping

#### Entire study area

The MTVF exhibited a strong ability to map cultivated land in the study area, and its OA, PA, UA, and kappa coefficient performance reached higher levels (accuracy > 90%, kappa coefficient > 0.85) (Table 6). Compared with other land types, the mapping accuracies (PA and UA) of the cultivated land were both the highest. The classification accuracies (PA and UA) of the grassland, woodland, and water bodies were also relatively high. The classification accuracies (PA and UA) of the artificial construction land and bare land were worse than those of the other land cover types. In particular, the UA of the artificial construction land was 73.95%, and the PA of the bare land was 61.67%. Thus, the classification of these land cover types was considered to have a poor accuracy (accuracy <75%). These land cover types may have been affected by the input features because the input features were selected specifically for cultivated land mapping.

**Table 6:** Accuracy assessment of the cultivated land mapping conducted using the method based on time series vector features (MTVF) for the entire study area.

Class	PA (%)	UA (%)
Cultivated land	99.41	97.66
Woodland	96.04	96.92
Grassland	84.37	92.46
Water	86.98	96.55
Artificial construction land	93.35	73.95
Bare land	61.67	91.70
**OA (%)**	90.22	
**Kappa coefficient**	0.8792	

#### Sub-study areas

In the four sub-study areas, the MTVF also yielded a strong cultivated land mapping accuracy (Table 7). The mapping accuracies of the cultivated land in the LZ and HB areas were the highest of the four sub-study areas, indicating the robustness of the method in distinguishing cultivated land from grassland and woodland. The UA and PA values of area XX were the lowest of the four sub-study areas, indicating that some of the cultivated land was incorrectly classified into other ground feature categories. The reason for the lower accuracy in area XX was that this area was also affected by dense and fragmented patterns and mixed pixels. The results for area WH show that the method proposed in this paper is affected by artificial orchard features to a certain extent.

**Table 7:** Accuracy assessment of cultivated land mapping using the MTVF in the sub-study areas.

Sub-study area	OA (%)	UA (%)	PA (%)	Kappa coefficient
LZ	99.95	99.9	99.89	0.9989
XX	98.83	89.54	98.7	0.9382
HB	99.72	99.27	99.89	0.9938
WH	97.64	96.54	99.66	0.9501

### Comparison with other classification models

**Fig 6:**
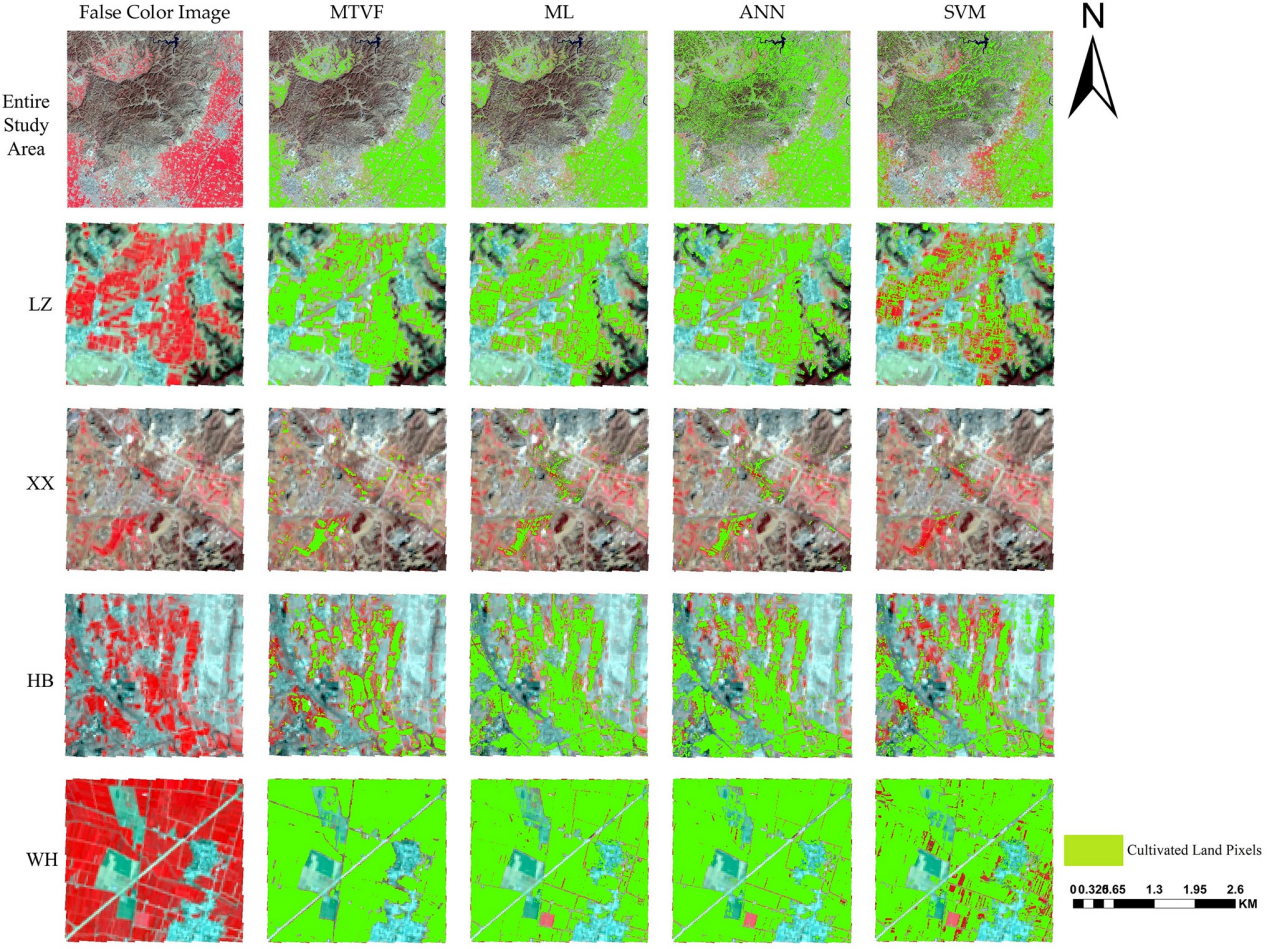
Comparison of cultivated land mapping for 2020. Note: the remote sensing image shown in this figure is a pseudo-color (R: NIR, G: Red, B: Green) Landsat 8 image acquired on March 19, 2020, and the cultivated land pixels are displayed in green. The image in Fig 6 is similar but not identical to the original figure used in the study, and therefore, it is presented for illustrative purposes only.

Fig 6 shows the difference between the cultivated land mapping results of the MTVF and the other models (i.e., the maximum likelihood (ML), support vector machine, and artificial neural network (ANN) models). From the perspective of the entire study area, the MTVF and ML had the best cultivated land mapping effects, and the large area of cultivated land located in a flat area could be distinguished with distinct boundaries. The difference between the cultivated land mapping capabilities of the MTVF and ML was mainly reflected in the mountainous areas (northwestern part of the entire study area), in which the ML contained more speckle noise, and much of the grassland was misclassified as cultivated land. In contrast, the ANN and SVM could not effectively filter out the cultivated land pixels in the study area, and the SVM had the worst cultivated land mapping ability. In the four sub-study areas, the SVM had the worst cultivated land mapping ability of all of the models and was not included in the subsequent comparison. In area LZ, the MTVF effectively distinguished several regular artificial forest land areas connected to cultivated land. Although the ANN and ML also exhibited this ability, some of the plantation pixels were still mistakenly classified as cultivated land. In area XX, broken patches and mixed pixels were the main factors that affected the accuracy of the cultivated land mapping. The ANN and ML had a limited ability to overcome these influencing factors. Therefore, the ANN and ML misclassified woodland and grassland in this sub-study area and generated more speckle noise. In area HB, the MTVF, ML, and ANN effectively avoided the influence of the grassland, but only the MTVF distinguished between the woodland pixels and cultivated land pixels. In the WH, the MTVF effectively avoided the impact of the roads between the cultivated land and retained wider country roads. However, the MTVF failed to completely avoid the impact of the orchards and the orchards were mistakenly classified as cultivated land.

The MTVF was compared with other classification models. From the perspective of the classification accuracy (Table 8), the MTVF, ANN, and ML all produced cultivated land maps with good accuracies. Their accuracies were greater than 75%, and their kappa coefficients were greater than 0.75. The cultivated land map generated by the SVM model had the worst accuracy. The SVM’s accuracy was not only the worst for the entire study area, but its accuracy performances in the four sub-study areas were also inferior to those of the other methods. In sub-study area XX, although the ANN achieved the highest OA and kappa coefficients, the MTVF achieved better UA and PA values. In general, the MTVF exhibited a better performance in cultivated land mapping than the other models.

**Table 8:** Comparison of the accuracies of the MTVF and the other classification models.

Sub-study area	Model	OA (%)	UA (%)	PA (%)	Kappa coefficient
Entire study area	MTVF	90.22	99.41	97.66	0.8792
	ANN	82.77	93.38	72.04	0.7888
	ML	79.82	98.62	95.36	0.7555
	SVM	73.01	64.2	52.91	0.6674
LZ	MTVF	99.95	99.9	99.89	0.9989
	ANN	98.25	98.57	97.51	0.9645
	ML	99.2	98.45	99.76	0.9838
	SVM	81.87	59.38	75.42	0.6176
XX	MTVF	97.43	98.04	99.75	0.8811
	ANN	98.83	89.54	98.7	0.9382
	ML	97.88	81.05	97.67	0.8836
	SVM	69.39	5.23	8.32	0.0892
HB	MTVF	99.72	99.27	99.89	0.9938
	ANN	98.21	95.36	99.37	0.9599
	ML	98.39	96.85	98.19	0.9633
	SVM	89.11	77.61	93.59	0.7647
WH	MTVF	97.64	96.54	99.66	0.9501
	ANN	93.64	90.53	83.96	0.8632
	ML	93.01	89.65	82.28	0.849
	SVM	90.13	86.81	98.7	0.7857

In this study, the statistics of the ground truth (pixels) in the confusion matrices of the cultivated land maps produced using the various methods were computed (Table 9). The cultivated land maps produced using the MTVF were the least affected by the other types of ground features. The impact of the woodland was dominant, but the artificial construction land and grassland also had impacts. Factors such as artificially planted forests in cultivated land, field ravines, and rural residential land boundaries were the reasons for the loss of accuracy of the MTVF. The ANN and SVM exhibited a weak ability to avoid the influence of the woodland, and the ANN model was also more susceptible to the influence of the grassland. In general, the forest land, grassland, and artificial construction land were the factors that affected the mapping of the cultivated land because there were often a large number of mixed pixels between the cultivated land in northern Henan and the above surface cover types, which led to misclassification. By limiting the impacts of these types of land, the MTVF achieved better results.

**Table 9:** Statistical comparison of the ground truth (pixels) of the MTVF and the other classification models.

Model	Cultivated land	Grassland	Woodland	Artificial construction land	Water	Bare land
MTVF	2674	5	63	12	0	0
ANN	2512	50	1084	16	3	0
ML	2653	14	128	24	0	0
SVM	1727	15	2469	15	1	0

Note: Cultivated land denotes the number of pixels that were correctly classified as cultivated land; and Grassland, Woodland, Artificial construction land, Water, and Bare land denote the sums of the number of pixels that were incorrectly classified as cultivated land and the cultivated land that was incorrectly classified as each category.

## Discussion

In this study, vector thinking was a core concept, and an MTVF that uses time series multispectral data for annual cultivated land mapping was developed. The feature extraction of the time series vectors was the core of this research. In this study, by introducing unit vectors, we extracted five features of each time series vector: *Max*_*p*_ (the maximum value inside vector p), *Min*_*p*_ (the minimum value inside vector p), *Ran*_*p*_ (the difference between *Max*_*p*_ and *Min*_*p*_), *Cos*_*p*_ (the cosine of the angle between time series vector p and the unit vector), and *Dis*_*p*_ (the distance between time series vector p and the unit vector). The features extracted from the different time series vectors effectively reduced the misclassification of land cover types caused by similar spectra and enlarged the differences between the land cover types and cultivated land.

In the MTVF, the selection of the features is very important. Selecting too few features cannot provide a sufficient classification basis for the classification model, and selecting too many features will lead to redundancy. In this study, the Gini coefficient was introduced to measure the importance of the different characteristics to the cultivated land mapping. Statistical analysis of the importance of each feature revealed that B12_Cos (importance score of 0.1723) and B8A_Dis (importance score of 0.1368) had the most significant Gini importance scores, indicating that the shortwave infrared band (Band 12) and the red edge band (Band 8A) made relatively large contributions to the cultivated land mapping. Among the vegetation index parameters, the NDVI_705__Min (importance score of 0.0526, ranked third) and ARVI_Cos (importance score of 0.0516, ranked fourth) had the highest importance scores. Traditional vegetation indices such as the normalized vegetation index (NDVI) and the enhanced vegetation index (EVI) did not have high importance scores in this study. This phenomenon revealed that non-traditional vegetation indices can also be valuable in cultivated land mapping, which is similar to the results of several previous studies [68, 69]. The parameters characterized by the vector angle and vector distance dominated the 10 most important parameters, indicating that spatial features based on time series vectors can reflect the differences between cultivated land and other land use types better than vector extreme value features. It should be noted that for different research areas, changes in the types of land cover will also lead to changes in the importance of the features. When conducting cultivated land mapping in different research areas, it is necessary to reassess the importance of these features [70].

In this study, the accuracy of the cultivated land mapping conducted using the MTVF was evaluated in the entire study area and in four sub-study areas. It was found that the cultivated land map obtained using the MTVF had the highest accuracy compared to the traditional classification models (i.e., the maximum likelihood, artificial neural network, and support vector machine models). The cultivated land map obtained using the MTVF had the lowest error and limited the effects of mixed pixels to a certain extent (Fig 6). However, the MTVF was still subject to interference from mixed pixels, resulting in some uncertainty in its ability to perform cultivate land mapping. In the study area, affected by the traditional farming practices in northern China, the distribution of cultivated land in some areas was irregular. There was cultivated land within villages and near mountain ravines, and trees were also planted in the cultivated land. The aforementioned areas with heterogeneous landscapes led to a large number of mixed pixels, resulting in errors in the mapping of the cultivated land [19, 71]. The spectral confusion between the woodland and cultivated land also contributed to the uncertainty of the cultivated land map obtained using the MTVF. In sub-study area WH, the spectral confusion between the cultivated land and orchards contributed to the misclassification of the MTVF to some extent (Fig 6).

## Conclusions

In this study, a simple and effective method for cultivated land mapping was developed. The MTVF has a stronger ability to eliminate the influences of other vegetation. By introducing vector thinking, an MTVF was developed. The cultivated land mapping performance of the MTVF was evaluated in the entire study area and in four sub-study areas, The main conclusions of this study are as follows.

1. The MTVF has a high potential for cultivated land mapping and achieved a high accuracy (greater than 90%) in the study area. The MTVF mainly mixed the effects of the pixels in the mapping of the cultivated land, especially where the land cover was complicated. However, in some cases, the MTVF also had the ability to limit the influence of the mixed pixels.
2. In terms of the importance scores, the shortwave infrared and red-edge bands of the Sentinel-2 satellite have a high potential for cultivated land mapping. The non-traditional vegetation indices were superior to the traditional vegetation indices in terms of cultivated land mapping. The spatial features based on time series vectors reflected the differences between cultivated land and other land use types better than the vector extreme features.
3. Compared with other models (i.e., the maximum likelihood, support vector machine, and artificial neural network models), the MTVF achieved the best results in the study area, but it still suffers interference from artificial woodland, field gullies, and rural settlement boundaries, which decrease the accuracy of the cultivated land map.

The MTVF provides a new method for cultivated land mapping. Future research should focus on combining this method with the mixed pixel decomposition algorithm. Combining multi-source sensor data (e.g., synthetic aperture radar) for use in cultivated land mapping should also be a future research focus because the mapping accuracy of the MTVF can still be affected by other ground cover types.

## References

[1] Xiao X., Boles S., Frolking S., Li C., Babu J. Y., Salas W., et al, “Mapping paddy rice agriculture in South and Southeast Asia using multi-temporal MODIS images,” *Remote sensing of Environment*. 100(1), 95–113 (2016) doi: 10.1016/j.rse.2005.10.004

[2] Mohammed I., Marshall M., de Bie K., Estes L., & Nelson A, “A blended census and multiscale remote sensing approach to probabilistic cropland mapping in complex landscapes,” *ISPRS journal of photogrammetry and remote sensing*. 161, 233–245 (2020) doi: 10.1016/j.isprsjprs.2020.01.024

[3] Justice, C., and Pierre Defourny, “Developing a strategy for global agricultural monitoring in the framework of Group on Earth Observations (GEO) Workshop Report,” *GEOSS Operational Agricultural Monitoring System*. (2007).

[4] Olofsson P., Stehman S. V., Woodcock C. E., Sulla-Menashe D., Sibley A. M., Newell J. D., et al, “A global land-cover validation data set, part I: Fundamental design principles,” *International Journal of Remote Sensing*. 33(18), 5768–5788 (2012) doi: 10.1080/01431161.2012.674230

[5] Pflugmacher D., Krankina O. N., Cohen W. B., Friedl M. A., Sulla-Menashe D., Kennedy R. E.,. et al, “Comparison and assessment of coarse resolution land cover maps for Northern Eurasia,” *Remote Sensing of Environment*. 115(12), 3539–3553 (2011) doi: 10.1016/j.rse.2011.08.016

[6] Giri C., Pengra B., Long J., & Loveland T. R, “Next generation of global land cover characterization, mapping, and monitoring,” *International Journal of Applied Earth Observation and Geoinformation*. 25, 30–37 (2013) doi: 10.1016/j.jag.2013.03.005

[7] Wu Z., Thenkabail P. S., Mueller R., Zakzeski A., Melton F., Johnson L., et al, “Seasonal cultivated and fallow cropland mapping using MODIS-based automated cropland classification algorithm,” *Journal of Applied Remote Sensing*. 8(1), 083685 (2014) doi: 10.1117/1.JRS.8.083685

[8] Delrue J., Bydekerke L., Eerens H., Gilliams S., Piccard I., & Swinnen E, “Crop mapping in countries with small-scale farming: A case study for West Shewa, Ethiopia,” *International journal of remote sensing*. 34(7), 2566–2582 (2013) doi: 10.1080/01431161.2012.747016

[9] Gong P., Wang J., Yu L., Zhao Y., Zhao Y., Liang L., et al, “Finer resolution observation and monitoring of global land cover: First mapping results with Landsat TM and ETM+ data,” *International Journal of Remote Sensing*. 34(7), 2607–2654 (2013) doi: 10.1080/01431161.2012.748992

[10] Yu L., Wang J., Clinton N., Xin Q., Zhong L., Chen Y., et al, “FROM-GC: 30 m global cropland extent derived through multisource data integration,” *International Journal of Digital Earth*. 6(6), 521–533 (2013) doi: 10.1080/17538947.2013.822574

[11] Chen J., Chen J., Liao A., Cao X., Chen L., Chen X.,. et al, “Global land cover mapping at 30 m resolution: A POK-based operational approach,” *ISPRS Journal of Photogrammetry and Remote Sensing*. 103, 7–27 (2015) doi: 10.1016/j.isprsjprs.2014.09.002

[12] Loveland T. R., Reed B. C., Brown J. F., Ohlen D. O., Zhu Z., Yang L, et al. Development of a global land cover characteristics database and IGBP DISCover from 1 km AVHRR data,” *International Journal of Remote Sensing*. 21(6–7), 1303–1330 (2000) doi: 10.1080/014311600210191

[13] Friedl M. A., McIver D. K., Hodges J. C., Zhang X. Y., Muchoney D., Strahler A. H., et al, “Global land cover mapping from MODIS: algorithms and early results,” *Remote sensing of Environment*. 83(1–2), 287–302 (2002) doi: 10.1016/S0034-4257(02)00078-0

[14] Friedl M. A., Sulla-Menashe D., Tan B., Schneider A., Ramankutty N., Sibley A., et al, “MODIS Collection 5 global land cover: Algorithm refinements and characterization of new datasets,” *Remote sensing of Environment*. 114(1), 168–182 (2010) doi: 10.1016/j.rse.2009.08.016

[15] Strahler A. H, “The use of prior probabilities in maximum likelihood classification of remotely sensed data,” *Remote sensing of Environment*. 10(2), 135–163 (1980) doi: 10.1016/0034-4257(80)90011-5

[16] Cortes C., & Vapnik V, “Support-vector networks,” *Machine learning*. 20(3), 273–297 (1995) doi: 10.1007/BF00994018

[17] Gong P., Pu R., & Chen J, “Mapping Ecological Land Systems and Classification Uncertainties from Digital Elevation and Forest-Cover Data Using Neural Networks,” *Photogrammetric Engineering & Remote Sensing*. 62, 1249–1260 (1996).

[18] Zhang D., Pan Y., Zhang J., Hu T., Zhao J., Li N., et al, “A generalized approach based on convolutional neural networks for large area cropland mapping at very high resolution,” *Remote Sensing of Environment*. 247, 111912 (2020) doi: 10.1016/j.rse.2020.111912

[19] Hao P., Löw F., & Biradar C, “Annual cropland mapping using reference Landsat time series—a case study in Central Asia,” *Remote Sensing*. 10(12), 2057 (2018) doi: 10.3390/rs10122057

[20] Maxwell A. E., Warner T. A., & Fang F, “Implementation of machine-learning classification in remote sensing: An applied review,” *International Journal of Remote Sensing*. 39(9), 2784–2817 (2018) doi: 10.1080/01431161.2018.1433343

[21] Ma L., Liu Y., Zhang X., Ye Y., Yin G., & Johnson B. A, “Deep learning in remote sensing applications: A meta-analysis and review,” *ISPRS journal of photogrammetry and remote sensing*. 152, 166–177 (2019) doi: 10.1016/j.isprsjprs.2019.04.015

[22] Vali A., Comai S., & Matteucci M, “Deep learning for land use and land cover classification based on hyperspectral and multispectral earth observation data: A review,” *Remote Sensing*. 12(15), 2495 (2020) doi: 10.3390/rs12152495

[23] Ortiz R., Sayre K. D., Govaerts B., Gupta R., Subbarao G. V., Ban T., et al, “Climate change: can wheat beat the heat?” *Agriculture*, *Ecosystems & Environment*. 126(1–2), 46–58 (2008) doi: 10.1016/j.agee.2008.01.019

[24] Potgieter A. B., Apan A., Hammer G., & Dunn P, “Early-season crop area estimates for winter crops in NE Australia using MODIS satellite imagery,” *ISPRS Journal of Photogrammetry and Remote Sensing*. 65(4), 380–387 (2010) doi: 10.1016/j.isprsjprs.2010.04.004

[25] Jonsson Per, and Eklundh Lars. "Seasonality extraction by function fitting to time-series of satellite sensor data." *IEEE transactions on Geoscience and Remote Sensing*. 40.8: 1824–1832 (2002) doi: 10.1109/TGRS.2002.802519

[26] Jia Kun, et al. "Land cover classification of Landsat data with phenological features extracted from time series MODIS NDVI data." *Remote sensing*. 6.11: 11518–11532 (2014) doi: 10.3390/rs61111518

[27] Suess Stefan, et al. "Characterizing 32 years of shrub cover dynamics in southern Portugal using annual Landsat composites and machine learning regression modeling." *Remote Sensing of Environment*. 219: 353–364 (2018) doi: 10.1016/j.rse.2018.10.004

[28] Henan Province Bureau of Statistics, *Henan Statistical Yearbook 2020*, China Statistics Press: Beijing, China (2021). (In Chinese)

[29] Luo D., Ye L., & Sun D, “Risk evaluation of agricultural drought disaster using a grey cloud clustering model in Henan province, China,” *International Journal of Disaster Risk Reduction*. 49, 101759 (2020) doi: 10.1016/j.ijdrr.2020.101759

[30] Kaiyong W., & Pengyan Z, “The Research on Impact Factors and Characteristic of Cultivated Land Resources Use Efficiency—take Henan Province, China as a Case Study,” *Ieri Procedia*. 5, 2–9 (2013) doi: 10.1016/j.ieri.2013.11.062

[31] Mather P.M, *Computer Processing of Remotely-Sensed Images*, 3rd ed., John Wiley & Sons, Ltd.:Chichester, UK (2004).

[32] Piper J, “Variability and bias in experimentally measured classifier error rates,” *Pattern Recognition Letters*. 13(10), 685–692 (1992) doi: 10.1016/0167-8655(92)90097-J

[33] Van Niel T. G., McVicar T. R., & Datt B, “On the relationship between training sample size and data dimensionality: Monte Carlo analysis of broadband multi-temporal classification,” *Remote sensing of environment*. 98(4), 468–480 (2005) doi: 10.1016/j.rse.2005.08.011

[34] Huete A. R, “A soil-adjusted vegetation index (SAVI),” *Remote sensing of environment*. 25(3), 295–309 (1988) doi: 10.1016/0034-4257(88)90106-X

[35] Baret F, Guyot G, Major DJ, “Crop biomass evaluation using radiometric measurements,” *Photogrammetria*. 43(5), 241–256 (1989) doi: 10.1016/0031-8663(89)90001-X

[36] Baret F., & Guyot G, “Potentials and limits of vegetation indices for LAI and APAR assessment,” *Remote sensing of environment*. 35(2–3), 161–173 (1991) doi: 10.1016/0034-4257(91)90009-U

[37] Qi J., Chehbouni A., Huete A. R., Kerr Y. H., & Sorooshian S, “A modified soil adjusted vegetation index,” *Remote sensing of environment*. 48(2), 119–126 (1994) doi: 10.1016/0034-4257(94)90134-1

[38] Tucker C. J, “Red and photographic infrared linear combinations for monitoring vegetation,” *Remote sensing of Environment*. 8(2), 127–150 (1979) doi: 10.1016/0034-4257(79)90013-0

[39] Major D. J., Baret F., & Guyot G, “A ratio vegetation index adjusted for soil brightness,” *International journal of remote sensing*. 11(5), 727–740 (1990) doi: 10.1080/01431169008955053

[40] Richardson A. J., & Wiegand C. L, “Distinguishing vegetation from soil background information,” *Photogrammetric engineering and remote sensing*. 43(12), 1541–1552 (1977).

[41] Crippen R E, “Calculating the vegetation index faster,” *Remote sensing of Environment*. 34(1), 71–73 (1990). doi: 10.1016/0034-4257(90)90085-Z

[42] Clevers, J. G. P. W, “The application of the weighted near-infrared-red vegetation index for estimating LAI at the vegetative and generative stage of cereals,” in Proc. 16th ISPRS-Congress, Kyoto, Japan (1998).

[43] Gitelson, A. A., Merzlyak, M. N., & Grits, Y, “Novel algorithms for remote sensing of chlorophyll content in higher plant leaves,” in IGARSS’96. 1996 International Geoscience and Remote Sensing Symposium (Vol. 4, pp. 2355–2357), IEEE, Lincoln, USA (1996).

[44] Pinty B., & Verstraete M. M, “GEMI: A Non-Linear Index to Monitoring Global Gegetation Index (MSAVI),” *Remote Sensing of Environment*. 48, 119–126 (1991). doi: 10.1007/BF00031911

[45] Kaufman Y. J., & Tanre D, “Atmospherically resistant vegetation index (ARVI) for EOS-MODIS,” *IEEE transactions on Geoscience and Remote Sensing*. 30(2), 261–270 (1992) doi: 10.1109/36.134076

[46] Delegido J., Verrelst J., Alonso L., & Moreno J, “Evaluation of sentinel-2 red-edge bands for empirical estimation of green LAI and chlorophyll content,” *Sensors*. 11(7), 7063–7081 (2011) doi: 10.3390/s110707063 22164004 PMC3231680

[47] Dash J., & Curran P. J, “Evaluation of the MERIS terrestrial chlorophyll index (MTCI),” *Advances in Space Research*. 39(1), 100–104 (2007) doi: 10.1016/j.asr.2006.02.034

[48] Daughtry C. S., Walthall C. L., Kim M. S., De Colstoun E. B., & McMurtrey J. E Iii, “Estimating corn leaf chlorophyll concentration from leaf and canopy reflectance,” *Remote sensing of Environment*. 74(2), 229–239 (2000) doi: 10.1016/S0034-4257(00)00113-9

[49] Frampton W. J., Dash J., Watmough G., & Milton E. J, “Evaluating the capabilities of Sentinel-2 for quantitative estimation of biophysical variables in vegetation,” *ISPRS journal of photogrammetry and remote sensing*. 82, 83–92 (2013) doi: 10.1016/j.isprsjprs.2013.04.007

[50] Castillo J. A. A., Apan A. A., Maraseni T. N., & Salmo S. G III, “Estimation and mapping of above-ground biomass of mangrove forests and their replacement land uses in the Philippines using Sentinel imagery,” *ISPRS Journal of Photogrammetry and Remote Sensing*. 134, 70–85 (2017) doi: 10.1016/j.isprsjprs.2017.10.016

[51] Blackburn G. A, “Quantifying chlorophylls and caroteniods at leaf and canopy scales: An evaluation of some hyperspectral approaches,” *Remote sensing of environment*. 66(3), 273–285 (1998) doi: 10.1016/S0034-4257(98)00059-5

[52] Rouse J. W., Haas R. H., Schell J. A., & Deering D. W, “Monitoring vegetation systems in the Great Plains with ERTS,” *NASA special publication*. 351(1974), 309 (1974).

[53] Evangelides Christos, and Nobajas Alexandre, “Red-Edge Normalised Difference Vegetation Index (NDVI_705_) from Sentinel-2 imagery to assess post-fire regeneration,” *Remote Sensing Applications*: *Society and Environment*. 17: 100283 (2020) doi: 10.1016/j.rsase.2019.100283

[54] Liu H. Q., & Huete A, “A feedback based modification of the NDVI to minimize canopy background and atmospheric noise,” *IEEE transactions on geoscience and remote sensing*. 33(2), 457–465 (1995) doi: 10.1109/TGRS.1995.8746027

[55] Xun L., Zhang J., Cao D., Zhang S., & Yao F, “Crop Area Identification Based on Time Series EVI2 and Sparse Representation Approach: A Case Study in Shandong Province, China,” *IEEE Access*. 7, 157513–157523 (2019) doi: 10.1109/ACCESS.2019.2949799

[56] Kruse Fred A., et al. "The spectral image processing system (SIPS)—interactive visualization and analysis of imaging spectrometer data." *Remote sensing of environment*. 44.2–3: 145–163 (1993) doi: 10.1016/0034-4257(93)90013-N

[57] Mars John C., and Rowan Lawrence C. "Spectral assessment of new ASTER SWIR surface reflectance data products for spectroscopic mapping of rocks and minerals." *Remote Sensing of Environment*. 114.9: 2011–2025 (2010) doi: 10.1016/j.rse.2010.04.008

[58] Lin Jian, et al. "A spatial-distance analysis approach of multi-spectrum feature distribution for remote sensing image land use/cover." *Spectroscopy and Spectral Analysis*. 29.2: 436–440 (2009). 19445222

[59] Swain Philip H., and Hauska Hans. "The decision tree classifier: Design and potential." *IEEE Transactions on Geoscience Electronics*. 15.3: 142–147 (1977) doi: 10.1109/TGE.1977.6498972

[60] Vieira Rita Marcia da Silva Pinto, et al. "Land degradation mapping in the MATOPIBA region (Brazil) using remote sensing data and decision-tree analysis." *Science of The Total Environment*. 782: 146900 (2021) doi: 10.1016/j.scitotenv.2021.146900

[61] de Colstoun Eric C. Brown, et al. "National Park vegetation mapping using multitemporal Landsat 7 data and a decision tree classifier." *Remote sensing of Environment*. 85.3: 316–327 (2003) doi: 10.1016/S0034-4257(03)00010-5

[62] Breiman L, “Random forests,” *Machine learning*. 45(1), 5–32 (2001) doi: 10.1023/A:1010933404324

[63] Pedregosa et al. “Scikit-learn: Machine Learning in Python,” JMLR 12. pp. 2825–2830 (2011).

[64] Li C., Wang J., Wang L., Hu L., & Gong P, “Comparison of classification algorithms and training sample sizes in urban land classification with Landsat thematic mapper imagery,” *Remote sensing*. 6(2), 964–983 (2014) doi: 10.3390/rs6020964

[65] Bazi Y., & Melgani F, “Toward an optimal SVM classification system for hyperspectral remote sensing images,” *IEEE Transactions on geoscience and remote sensing*. 44(11), 3374–3385 (2006) doi: 10.1109/TGRS.2006.880628

[66] Atkinson P. M., & Tatnall A. R, “Introduction neural networks in remote sensing,” *International Journal of remote sensing*. 18(4), 699–709 (1997).

[67] Ji C. Y, “Land-use classification of remotely sensed data using Kohonen self-organizing feature map neural networks” *Photogrammetric engineering and remote sensing*. 66(12), 1451–1460 (2000).

[68] Zhang Lifu, et al. "Monitoring vegetation dynamics using the universal normalized vegetation index (UNVI): An optimized vegetation index-VIUPD." *Remote Sensing Letters*. 10.7: 629–638 (2019). doi: 10.1080/2150704X.2019.1597298

[69] da Silva Vanessa Sousa, et al. "Methodological evaluation of vegetation indexes in land use and land cover (LULC) classification." *Geology*, *Ecology*, *and Landscapes*. 4.2: 159–169 (2020). doi: 10.1080/24749508.2019.1608409

[70] Mróz Marek, and Sobieraj Anna. "Comparison of several vegetation indices calculated on the basis of a seasonal SPOT XS time series, and their suitability for land cover and agricultural crop identification." *Technical sciences*. 7.7: 39–66 (2004).

[71] Löw Fabian, and Duveiller Grégory. *"* Defining the spatial resolution requirements for crop identification using optical remote sensing." *Remote Sensing*. 6.9: 9034–9063 (2014). doi: 10.3390/rs6099034

